# SDF-1/CXCR7 axis regulates the proliferation, invasion, adhesion, and angiogenesis of gastric cancer cells

**DOI:** 10.1186/s12957-016-1009-z

**Published:** 2016-10-06

**Authors:** De-Min Ma, Dian-Xi Luo, Jie Zhang

**Affiliations:** 1 Department of Hepatobiliary and Vascular Surgery, People’s Hospital of Dezhou, Dezhou, Shandong Province 253014 People’s Republic of China; 2 Department of Gastrointestinal Surgery, People’s Hospital of Dezhou, 1751 Xin Hu Road, Dezhou, Shandong Province 253014 People’s Republic of China

**Keywords:** Gastric cancer, Chemokines, Stromal cell-derived factor-1, CXC chemokine receptor-7, Metastasis

## Abstract

**Background:**

More recent studies have revealed that chemokine receptor CXCR7 plays an important role in cancer development. However, little is known about the effect of CXCR7 on the process of gastric cancer cell invasion and angiogenesis. The aim of this study is to investigate the expression of CXCR7 in gastric cancer cell lines and to evaluate the role of CXCR7 in the proliferation, invasion, adhesion, and angiogenesis of gastric cancer cells.

**Methods:**

Real-time PCR and Western blotting were used to examine the mRNA and protein levels of CXCR4 and CXCR7 in five gastric cancer cell lines (HGC-27, MGC-803, BGC-823, SGC-7901, and MKN-28). CXCR7-expressing shRNA was constructed and subsequently stably transfected into the human gastric cancer cells. In addition, the effect of CXCR7 inhibition on cell proliferation, invasion, adhesion, VEGF secretion, and tube formation was evaluated.

**Results:**

The mRNA and protein of CXCR7 were expressed in all five gastric cancer cell lines; in particular, the expression of CXCR7 was the highest in SGC-7901 cells. Stromal cell-derived factor-1 (SDF-1) was found to induce proliferation, invasion, adhesion, and tube formation. Moreover, the VEGF secretion in SGC-7901 cells was also enhanced by SDF-1 stimulation. These biological effects were inhibited by the silencing of CXCR7 in SGC-7901 cells.

**Conclusions:**

Increased CXCR7 expression was found in gastric cancer cells. Knockdown of CXCR7 expression by transfection with CXCR7shRNA significantly inhibits SGC-7901 cells’ proliferation, invasion, adhesion, and angiogenesis. This study provides new insights into the significance of CXCR7 in the invasion and angiogenesis of gastric cancer.

## Background

Gastric cancer is one of the most commonly diagnosed malignancies and the main cause of cancer-related deaths in Asian populations [1]. Most deaths from gastric cancer are caused by metastasis, of which lymph node metastasis is the most common cause, which leads to the failure of surgery, chemotherapy, or radiotherapy [2]. Therefore, inhibition of metastatic gastric cancer is an important therapeutic strategy. However, the molecular mechanisms involved in this process have not been fully elucidated.

Stromal cell-derived factor-1 (SDF-1, also called CXCL12) is a member of the CXC subfamily of chemokines and is expressed in a variety of tissues including the lung, liver, bone marrow, and lymph nodes [3–5]. SDF-1 elicits biologic function through binding to its receptor, CXCR4, which is present on the cell surface and is a seven-transmembrane span G-protein-coupled receptor [6]. SDF-1 plays a role in a number of important physiological processes including leukocyte trafficking and vasculogenesis [7, 8]. More importantly, SDF-1 plays a crucial role in the process of invasion and metastasis of tumor cells [9]. SDF-1 stimulates proliferation, dissociation, migration, and invasion in a wide variety of tumor cells, including breast cancer cells [10], pancreatic cancer cells [11] and HCC cells [12], and gastric cancer cells [13].

Until recently, CXCR4 was considered to be the only receptor for SDF-1. However, a recent study has shown that chemokine receptor CXC chemokine receptor type 7 (CXCR7) can also bind to SDF-1, and it is identified as a second receptor for SDF-1 [14]. It has been demonstrated that CXCR7 is expressed in a variety of tumor cell lines and normal cells including activated endothelial cells, fetal liver cells, T cells, B cells, and renal multipotent progenitors [14, 15]. However, the function of CXCR7 is still unclear and controversial. Some studies suggested that CXCR7 is a non-signaling decoy receptor and cannot activate intracellular signaling cascades, while others considered that CXCR7 was a signaling receptor and could activate (mitogen-activated protein kinase (MAPK)) p42/44 and AKT phosphorylation through binding with SDF-1 [16, 17].

There is increasing evidence that CXCR7 may participate in tumor development. On the one hand, overexpression of CXCR7 has been observed in various tumors, including breast cancer, lung cancer, prostate cancer, and pancreatic cancer [18, 19]. On the other hand, expression of CXCR7 enhances the tumor cells’ proliferation, adhesion, invasion, and blood vessel sprout formation in vitro and promotes tumor growth in vivo [20, 21]. Although the role of SDF-1 in the promotion of invasive growth is well documented and the intracellular signals triggered by CXCR4 activation have been extensively investigated [22, 23], the role of SDF-1/CXCR7 axis in regulating tumor growth of gastric cancer is not yet known.

Therefore, the present study was undertaken to test the hypothesis that SDF-1/CXCR7 was involved in malignant properties of gastric cancer cells. We have studied the expression of CXCR7 in gastric cancer cell lines. We have also evaluated the effect of specific inhibition of CXCR7 on SDF-1-induced cell invasion, adhesion, and angiogenesis.

## Methods

### Cell culture

Human gastric cancer cell lines (HGC-27, MGC-803, BGC-823, SGC-7901, and MKN-28) and human umbilical vein endothelial cells (HUVECs) were purchased from Cell Bank of Shanghai Institute of Biochemistry and Cell Biology, Chinese Academy of Sciences (Shanghai, China). Gastric cancer cell lines were grown in Roswell Park Memorial Institute (RPMI) 1640 medium (Sigma-Aldrich, USA) that contained 10 % fetal bovine serum (FBS; HyClone, USA). HUVECs were maintained in DMEM medium containing 10 % FBS. All the media were supplemented with 100 U/ml penicillin and 100 μg/ml streptomycin (Invitrogen, USA) and maintained in 5 % CO_2_ at 37 °C.

### Construction of small hairpin RNA plasmid

Knockdown of CXCR7 was achieved by expression of short hairpin RNA (shRNA) from the pGPU6/Neo vector containing the human U6 promoter (GenePharma, Shanghai, China). The sequence of the oligonucleotide targeted to CXCR7 is 5′-GCATCTCTTCGACTACTCAGA-3′, corresponding to positions 223 to 243 within the CXCR7 mRNA sequence (accession no. NM_020311). The following complementary oligonucleotide encoding shRNA was designed to knock down CXCR7: (sense) 5′-CACCGCATCTCTTCGACTACTCAGATTCAAG AGATCTGAGTAGTCGAAGAGATGCTTTTTTG-3′ and (antisense) 5′-GATCCAAAAAAGCATCTCTTCGACTACTCAGATCTCTTGAATCTGAGTAGTCGAAGAGATGC-3′. The pGPU6/Neo plasmid was linearized with BamHI and BbsI to permit the insertion of the annealed oligonucleotides. DNA oligonucleotides were annealed by incubating the mixed oligonucleotides in the PCR thermocycler using the following profile: 95 °C for 5 min, 80 °C for 5 min, and 75 °C for 5 min, and gradually cooled to room temperature. Annealed oligonucleotides were ligated to the BbsI and BamHI sites of the pGPU6/Neo plasmid. The scrambled shRNA was used as a negative control (referred to as “NC” in the text), of which the sequence was 5′-GACGAGCTTCTACACAATCAT-3′. The recombinant constructs were verified by DNA sequencing and by analyzing the fragments generated from digestion with BamHI.

### Generation of stable transfectants

SGC-7901 cells were seeded in six-well plates to 80–90 % confluence. The cells were transfected with mixtures of shRNA plasmids and Lipofectamine™ 2000 reagent (Invitrogen, USA) according to the manufacturer’s instructions. Forty-eight hours after transfection, the transfected cells were grown in a growth medium containing 0.4 mg/ml G418 (Gibco, USA) for selection. Stable transfectant clones with low expression of CXCR7 were evaluated by real-time PCR (RT-PCR) and Western blot analysis. Stable transfectants were expanded for subsequent experiments. SGC-7901 cells transfected by CXCR7shRNA were referred to as CXCR7shRNA cells, while SGC-7901 cells transfected by scrambled shRNA as NC cells.

### Real-time PCR

Total RNA from gastric cancer cells was isolated using TRIzol (Invitrogen, Carlsbad, CA, USA) and then reverse transcribed with PrimeScript RT Master Mix (Takara, Otsu, Japan). RT-PCR was conducted using an Eppendorf Mastercycler ep realplex machine (Eppendorf, Germany) and using SYBR Premix Ex Taq™ II Kit (Takara) according to the manufacturer’s instructions. The primers were as follows: CXCR7, forward (5′-TGGGTGGTCAGTCTCGT-3′) and reverse (5′-CCGGCAGTAGGTCTCAT-3′); CXCR4, forward (5′-CCTGAAGTACCCCATCGAGCAC-3′) and reverse (5′-ATACCCCCTCGTAGATGGGCACA-3′); GAPDH, forward (5′-GAAGGTGAAGGTCGGAGTC-3′) and reverse (5′-GAAGATGGTGATGGGATTTC-3′). Relative mRNA expression levels were calculated by the 2^-△△Ct^ method. GAPDH was used as a reference gene.

### Western blot

For the preparation of lysates, the cells were washed with ice-cold PBS solution and lysed in lysis buffer. Cells were scraped into microcentrifuge tubes and centrifuged at 10,000×*g* for 15 min at 4 °C. The supernatant was collected, and protein concentrations were determined with the BCA assay kit (Sigma-Aldrich, USA) according to the manufacturer’s instruction. Samples were subjected to 10 % PAGE analysis after they were boiled for 5 min and electrophoretically transferred to polyvinylidene difluoride (PVDF) membranes (Millipore, USA). Blocking was performed in 5 % nonfat dried milk in Tris-buffered saline containing 0.1 % Tween 20 at room temperature for 1 h. Membranes were then incubated with primary antibody under constant agitation at antibody dilutions suggested by the antibody supplier overnight at 4 °C. After several washings, membranes were incubated with horseradish peroxidase-conjugated secondary antibody (anti-rabbit) for 1 h at room temperature under constant agitation. Proteins were visualized by using an enhanced chemiluminescence system (ECL; Amersham Biosciences, USA).

### Immunoprecipitation

Total protein extracts in a final volume of 250 ml were incubated overnight at 4 °C with 5 μg rabbit anti-CXCR7 and 5 μg rabbit anti-SDF-1 antibodies, previously bound to protein G magnetic beads (Millipore). An irrelevant rabbit polyclonal antibody bound to protein G magnetic beads was performed as a negative control. The immune complexes were precipitated by placing the tube into the magnetic stand (Millipore) and washing three times with 500 μL of PBS containing 0.1 % Tween 20. Precipitated proteins were separated by SDS-PAGE and analyzed by Western blotting with mouse anti-CXCR7 or mouse anti-SDF-1 antibody.

### Cell proliferation assay

SGC-7901 cells (including control, NC, and CXCR7shRNA transfected groups) were seeded into 96-well plates at a density of 5 × 10^3^ cells per well without FBS. After 24 h, the cultures were washed and re-fed with medium that contained SDF-1 (100 ng/ml; Peprotech, UK). After different time points (24, 48, 72, and 96 h), the number of viable cells was counted using a CCK8 assay (KeyGen, China) according to the manufacturer’s instructions. The quantity of formazan product measured at 490 nm was proportional to the number of live cells in the culture. The experiments were repeated in triplicates.

### Cell invasion assay

SGC-7901 cell invasion in response to SDF-1 was assayed in the Biocoat Matrigel invasion chamber (Becton Dickinson, USA) with 8-μm porosity polycarbonate filter membrane that was coated with Matrigel. SGC-7901 cells were suspended at 3 × 10^5^ cells/ml in serum-free media, respectively, and then 0.2 ml cell suspension was added to the upper chamber. Next, 0.5 ml serum-free media with SDF-1 (100 ng/ml) was added to the lower chamber. The chambers were then incubated for 24 h at 37 °C with 5 % CO_2_. After incubation, noninvasive cells were gently removed from the top of the Matrigel with a cotton-tipped swab. Invasive cells at the bottom of the Matrigel were fixed in 4 % paraformaldehyde and stained with hematoxylin. The number of invasive cells was determined by counting the hematoxylin-stained cells. For quantification, cells were counted under a microscope in five fields.

### Cell adhesion assay

Cell adhesion assay was carried out by using the CytoSelect™ ECM Cell Adhesion Assay kit (Cell Biolabs Inc., USA) following the instruction manual. Briefly, the 48-well plate precoated with laminin (LN) or fibronectin (FN) were washed with PBS twice and blocked for 1 h at 37 °C with RPMI 1640 containing 0.1 % bovine serum albumin (BSA) before plating cells. Plates were again washed with PBS and air dried. SGC-7901 cells were preincubated with SDF-1 (100 ng/ml) for 24 h at 37 °C. A cell suspension containing 2 × 10^5^ cells/ml was prepared in serum-free media. The cell suspension (150 μl) was added to the inside of each well (BSA-coated wells were provided as a negative control). Cells were allowed to attach for 1 h at 37 °C. Subsequently, unattached cells were removed by gentle washing three times with PBS. Then, the attached cells were stained with 1 % crystal violet. Each well was gently washed three times with PBS. The total crystal violet bound to the cells was eluted with 10 % acetic acid and measured by the absorbance at 560 nm. All the experiments were repeated three times in duplicate wells.

### In vitro tube formation coculture assay

The ability of endothelial cells to align into tube-like structures was measured using Matrigel™ tube formation assay as described previously [24]. Briefly, Transwell chambers were precoated with growth factor-reduced Matrigel (200 μL of 10 mg/mL). Control, NC, and CXCR7shRNA transfected SGC-7901 cells were seeded at a density of 2 × 10^4^ cells/well in 24-well plates and cultured for 24 h, respectively. HUVECs (2 × 10^4^ cells/well) were then seeded in Transwell chambers precoated with the Matrigel. Subsequently, Transwell chambers containing HUVECs were inserted into the 24-well plates and cocultured for 24 h. After 24 h of cocultured at 37 °C and 5 % CO_2_, the number of capillary-like tubes from three randomly chosen fields was counted and photographed under an Nikon inverted microscope (Japan). Tubes were defined as endothelial cells that had aligned to form >90 % closed structures [25].

### ELISA for VEGF

SGC-7901 cells were plated in 24-well tissue culture plates at a density of 1 × 10^5^ cells per well and followed with serum starvation for 24 h with RPMI 1640. Then, cells were treated with recombinant human SDF-1 (100 ng/ml), and the supernatants were collected 24 h after treatment. Vascular endothelial growth factor (VEGF) concentration was determined using Quantikine ELISA Kits according to the manufacturer’s instructions (R&D Systems, Minneapolis, MN).

### Statistical analysis

Data are reported as means ± SD. The one-way ANOVA was used for data analysis. All statistics were calculated using SPSS 16.0 software (SPSS, Chicago, IL, USA). *P* < 0.05 was considered as statistically significant.

## Results

### Expression of CXCR7 on gastric cancer cell lines and HUVECs

**Fig. 1:**
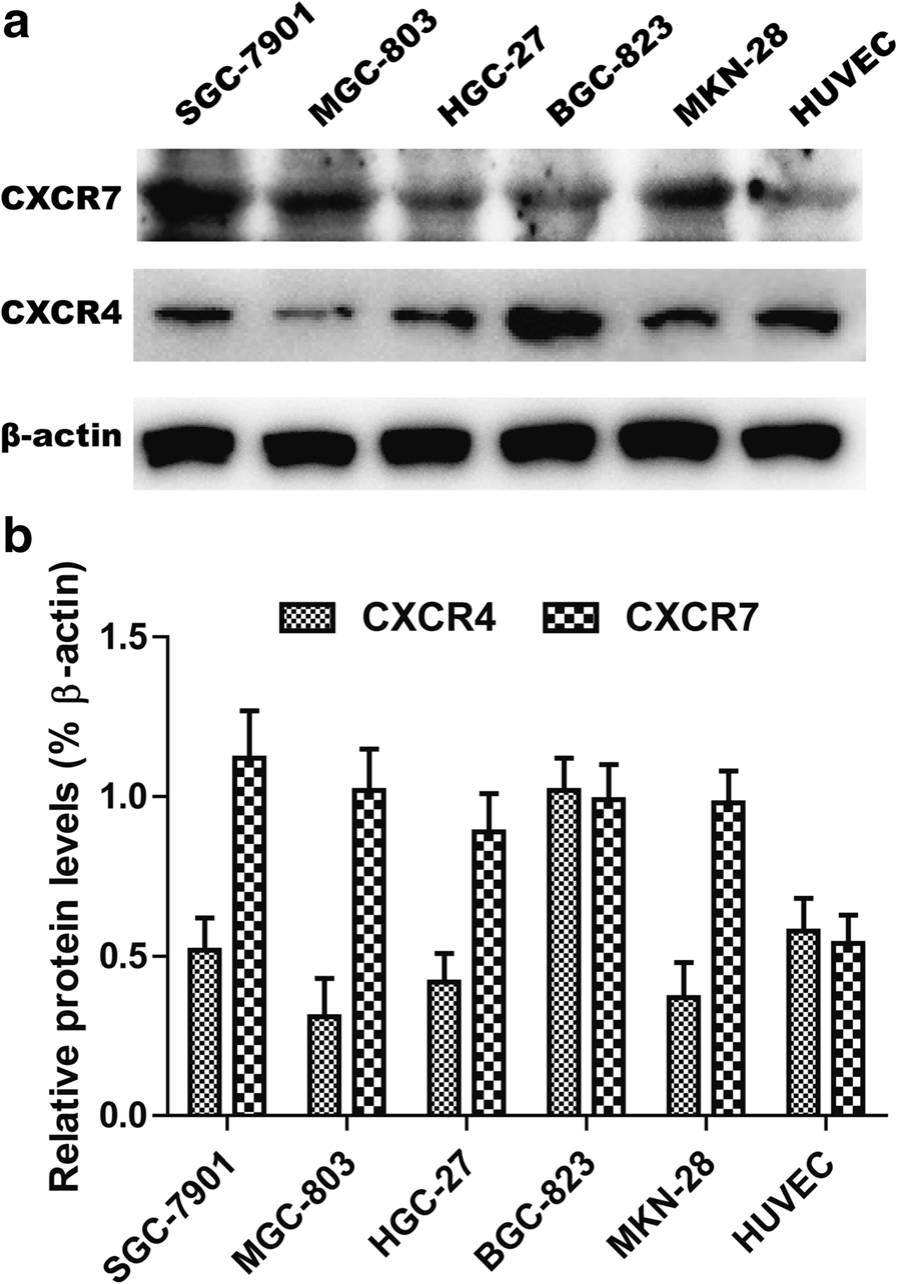
Expression of CXCR4 and CXCR7 in gastric cancer cell lines and HUVECs. **a** Western blot analysis was performed to detect CXCR7 and CXCR4 protein expression. β-Actin was used as a control to ensure equal loading. **b** RT-PCR was performed on various cell lines to determine CXCR7 and CXCR4 mRNA expression. GAPDH was used as a control. Data shown is representative of means ± SD of three independent experiments

To determine whether CXCR7 is expressed in gastric cancer cell lines, we first evaluated the expression of CXCR7 by Western blot in a panel of gastric cancer cell lines (HGC-27, MGC-803, BGC-823, SGC-7901, and MKN-28) and HUVEC. As shown in Fig. 1, CXCR7 protein expression was clearly detected in five gastric cancer cell lines and HUVEC, with different amounts of CXCR7 transcripts; in particular, the expression of CXCR7 was the highest in SGC-7901 cells.

### Interaction between CXCR7 and SDF-1 in SGC-7901 cells

**Fig. 2:**
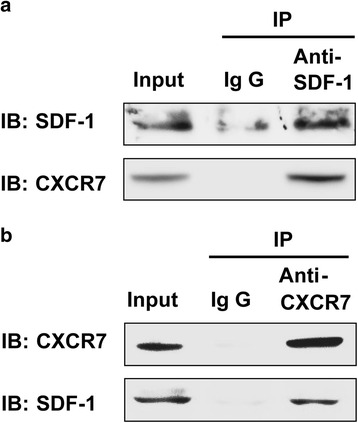
SDF-1 interacts with CXCR7 in SGC-7901 cells. Whole cell extracts from SGC-7901 cells were immunoprecipitated with a **a** rabbit anti-CXCR7 antibody and **b** rabbit anti-SDF-1 antibody. An irrelevant antibody (IgG) was used as control. Immunoprecipitated proteins were analyzed by Western blotting with mouse anti-CXCR7 and anti-SDF-1 antibodies

In order to prove the interaction between CXCR7 and SDF-1 in SGC-7901 cells, the total protein extracts from SGC-7901 cells were immunoprecipitated with an anti-CXCR7 or anti-SDF-1 antibody, precipitated proteins were analyzed by immunoblotting with antibodies directed specifically to either CXCR7 or SDF-1. Figure 2a showed that SDF-1 was pulled down together with CXCR7 by the anti-CXCR7 antibody in SGC-7901 cells, whereas none of these two proteins was recovered when an irrelevant antibody (IgG0) was used for immunoprecipitation, thus establishing the specificity of the assays. In Fig. 5b, CXCR7 was pulled down together with rabbit anti-SDF-1 antibody. Co-immunoprecipitation with each specific antibody proved association between CXCR7 and SDF-1 in SGC-7901 cells, which proved the formation of SDF-1/CXCR7 protein complex in SGC-7901 cells.

### The CXCR7shRNA causes effective and specific downregulation of CXCR7 expression

**Fig. 3:**
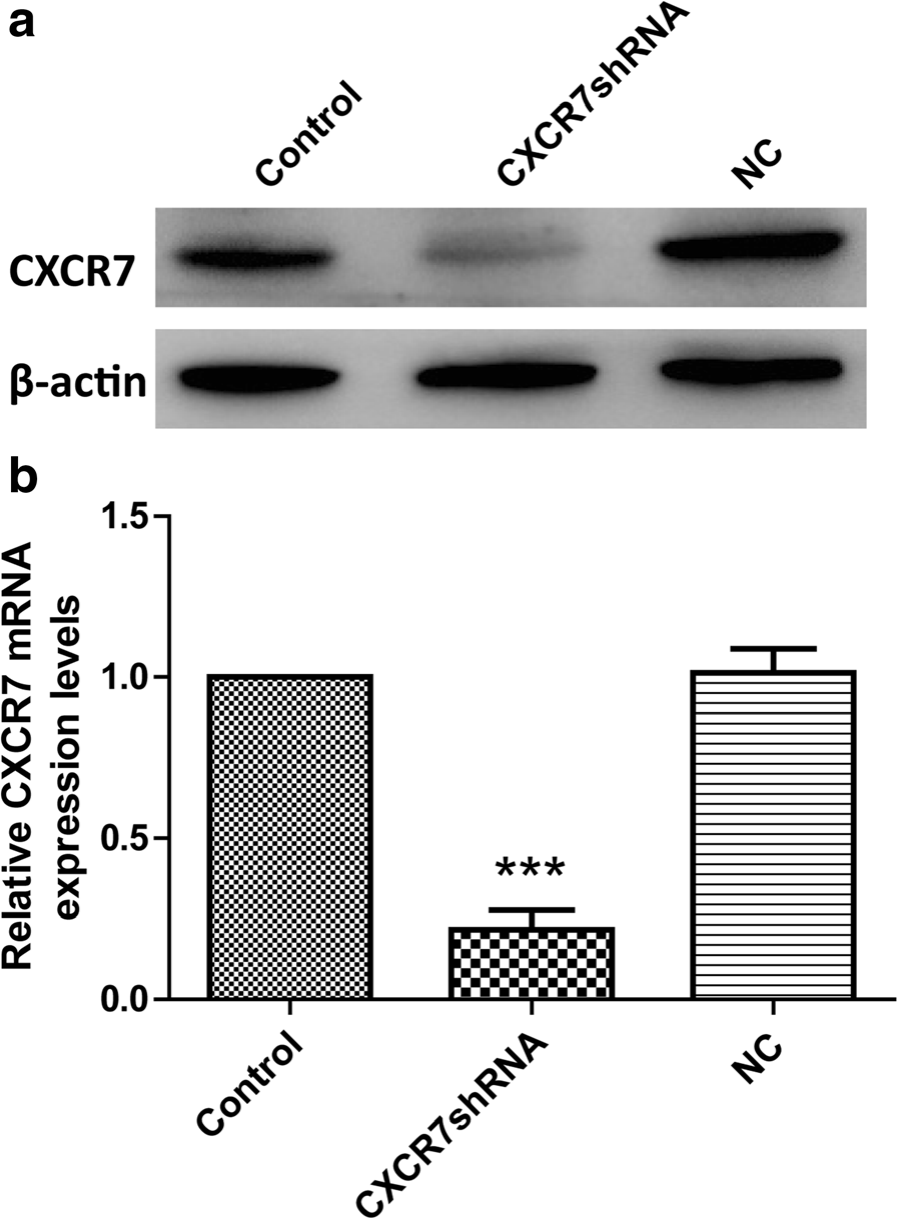
Downregulation of CXCR7 expression in SGC-7901 cells by transfection with CXCR7shRNA. **a** After G418 selection, the protein expression levels of CXCR7 were measured by Western blot using anti-CXCR7 antibody and β-actin as a loading control. The experiment was repeated three times with similar results. **b** Cellular RNA was harvested after G418 selection, and CXCR7 mRNA was measured using RT-PCR. GAPDH was used as a loading control

In order to study the potential role of CXCR7 in gastric cancer cell lines, CXCR7shRNA and scrambled shRNA were used to transfect SGC-7901 cells. After G418 selection, the knockdown efficiencies were subsequently tested using RT-PCR and Western blot. As shown in Fig. 3b, CXCR7 mRNA levels were reduced by 80 % in CXCR7shRNA-transfected cells, compared with the control cells. Similar to the RT-PCR results, the expression level of CXCR7 protein was significantly reduced in CXCR7shRNA-transfected cells (Fig. 3a). The scrambled sequence shRNA had no effect on CXCR7 expression. These results demonstrated that the expression of CXCR7 was specifically silenced in SGC-7901 cells.

### CXCR7 silencing inhibits SDF-1-induced SGC-7901 cells proliferation in vitro

**Fig. 4:**
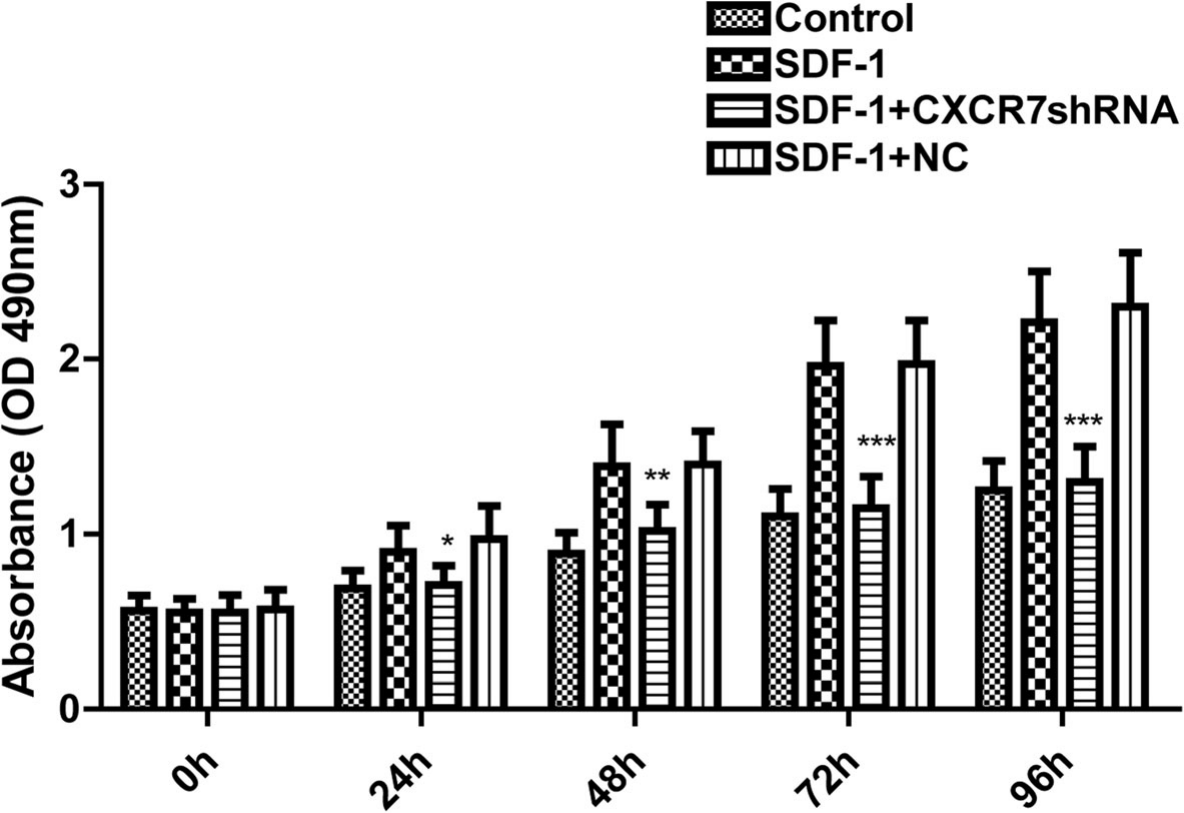
Silencing of CXCR7 inhibits SDF-1-induced enhancement on SGC-7901 cell proliferation in vitro. Cell proliferation was measured by CCK-8 at different time points (0, 24, 48, 72, and 96 h). Each *bar* represents mean ± SD from three independent experiments. **P < 0.01 (as compared with untransfected cells)

To evaluate a role of CXCR7 in regulating the proliferation of tumor cells, we selected the SGC-7901 cell line as a model. After incubation for 24 to 96 h, cell proliferation was significantly enhanced by SDF-1 (Fig. 4). We next evaluated the effect of silencing of CXCR7 on SGC-7901 cells proliferation. The CXCR7shRNA cells displayed decreased proliferation ability compared with the control cells and NC cells (Fig. 4). Taken together, these findings indicate that SDF-1 potently enhances the proliferation ability of SGC-7901 cells and that silencing of CXCR7 inhibits the proliferation ability of the cells induced by SDF-1.

### CXCR7 silencing inhibits SDF-1-induced SGC-7901 cell invasion in vitro

**Fig. 5:**
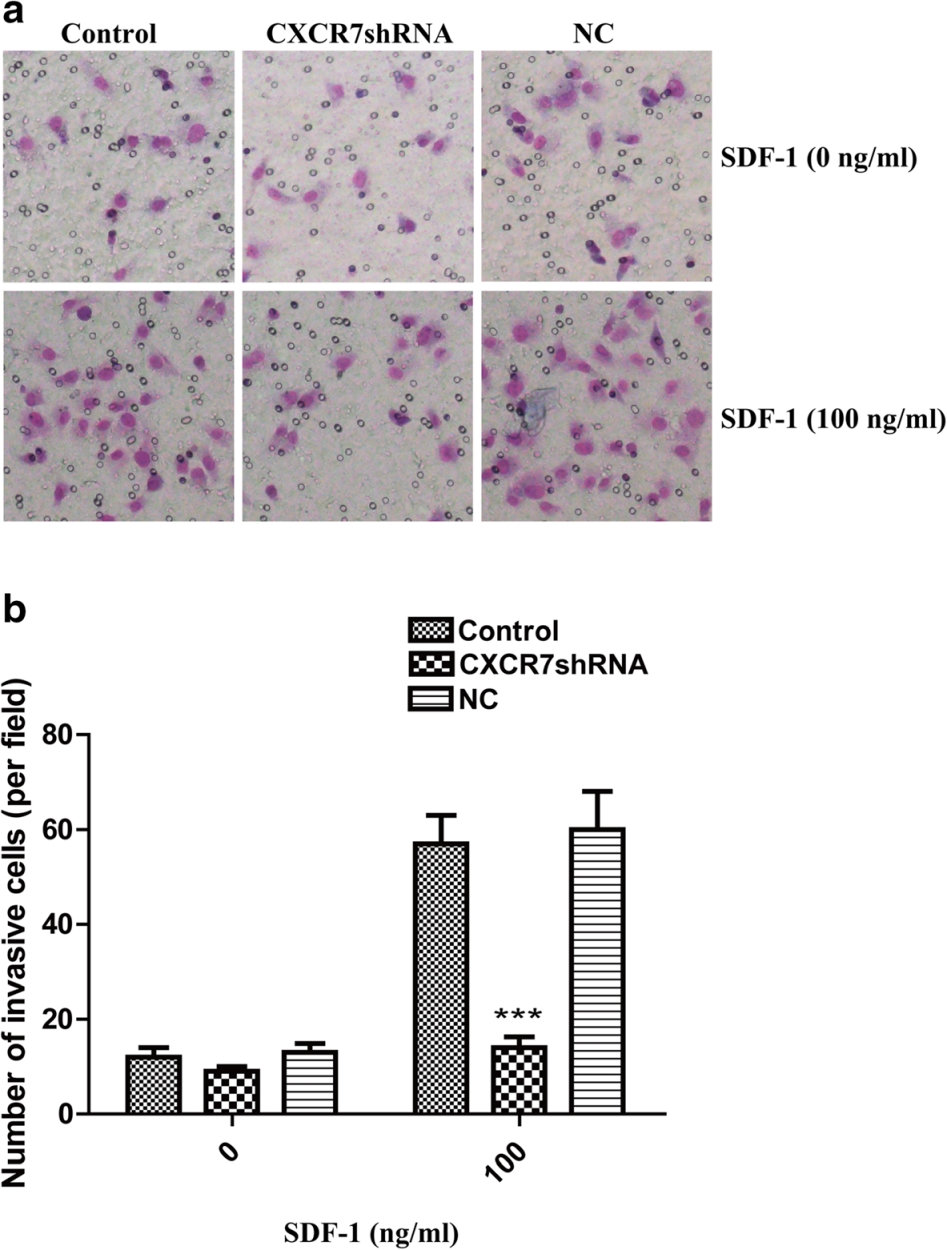
Silencing of CXCR7 inhibits SDF-1-induced enhancement on SGC-7901 cell invasion in vitro. **a** CXCR7shRNA transfected, NC, and control cells were treated with and without SDF-1 (0 or 100 ng/ml). **b** Mean number of invasive cells from five independent fields/well is indicated. Data are expressed as means ± SD from three independent experiments. ***P < 0.001 (as compared with control cells)

Cell invasion experiments were performed with a Matrigel invasion chamber, which is considered an in vitro model system for metastasis. As shown in Fig. 5, SGC-7901 cells spontaneously invaded through artificial basement membrane in the absence of SDF-1. In addition, we found that SDF-1-induced a significant increase of cancer cell invasion through Matrigel. We next evaluated the effect of silencing of CXCR7 on SGC-7901 cells invasion. The CXCR7shRNA cells displayed decreased invasive ability compared with the control cells and NC cells (Fig. 5). Taken together, these findings indicate that SDF-1 potently enhances the invasive ability of SGC-7901 cells and that silencing of CXCR7 inhibits the invasive behavior of the cells induced by SDF-1.

### CXCR7 silencing inhibits SDF-1-induced SGC-7901 cell adhesion in vitro

**Fig. 6:**
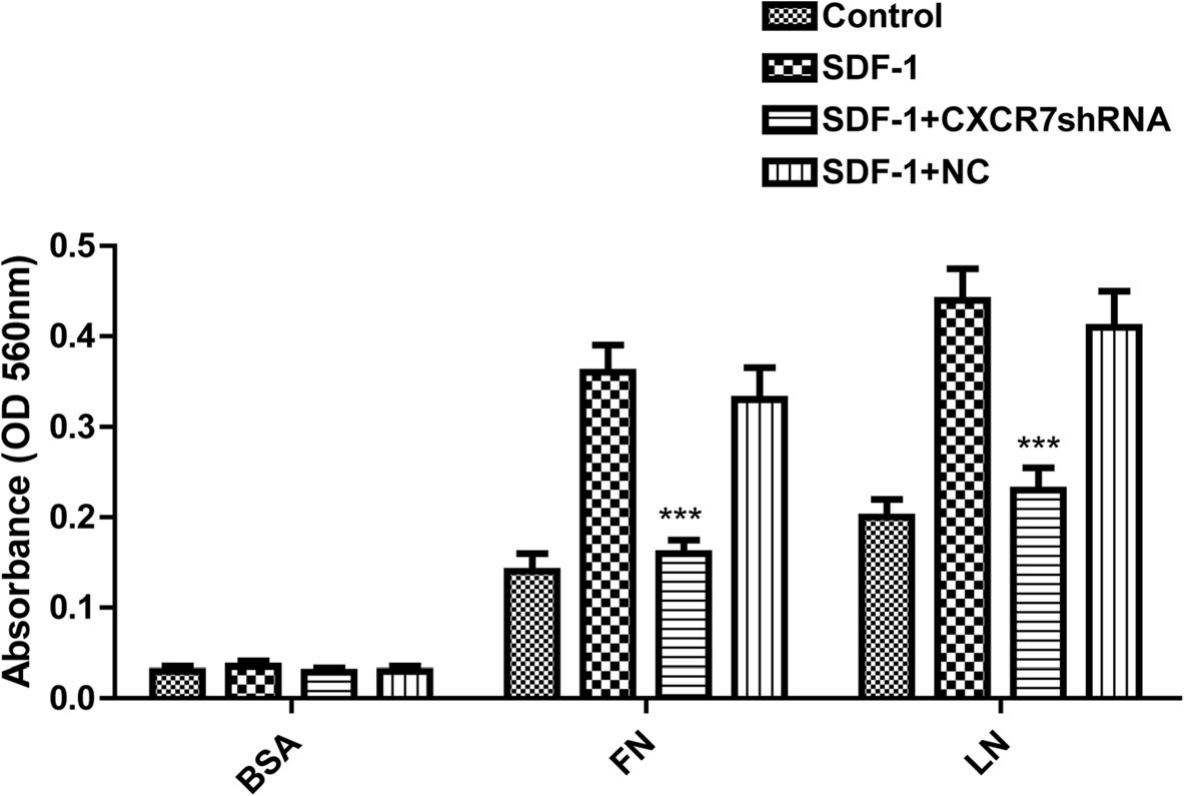
Effect of CXCR7 silencing on SGC-7901 cell adhesion in vitro. SGC-7901 cells were treated as described in the “Methods” section. Each *bar* represents mean ± SD from three independent experiments. ***P < 0.05 (as compared with untransfected cells)

To analyze the effect of CXCR7 expression on the adhesion of tumor cells to LN or FN, SGC-7901 cells were examined by a cell adhesion assay. As shown in Fig. 6, SGC-7901 cells displayed an enhanced cell adhesion to LN or FN in the presence of SDF-1. Adhesion of SGC-7901 cells to LN was greater than adhesion of SGC-7901 to FN or BSA. However, cells transfected by CXCR7shRNA showed significantly reduced ability of adhesion to LN or FN compared with the control and NC cells. Control, NC, and CXCR7shRNA transfected cells adhered equally to BSA-coated dishes. Together, these results indicate that treatment with SDF-1 increases the adhesive ability of SGC-7901 cells and CXCR7 silencing results in decreased adhesive ability.

### CXCR7 silencing inhibits SGC-7901 cell-induced tube formation in vitro

**Fig. 7:**
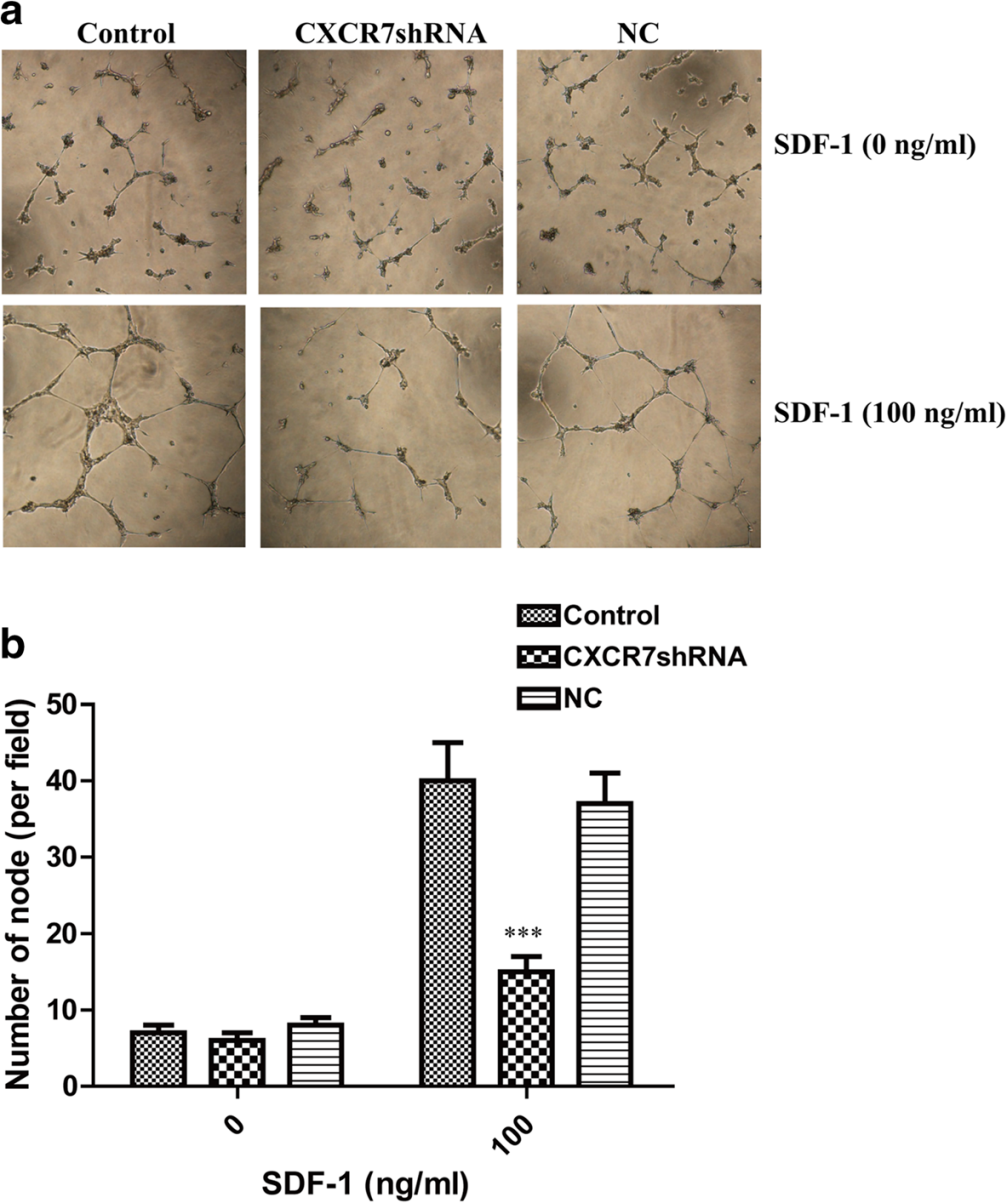
Effect of CXCR7 silencing on tube formation induced by SGC-7901 cells. HUVECs were cocultured with SGC-7901 cells, as described in the “Methods” section. **a** Representative images of tube-like structures are given for control, NC, and CXCR7shRNA transfected SGC-7901 cells with or without SDF-1. **b** Quantitative analysis of the number of tubes. Each *bar* represents mean ± SD from three independent experiments. ***P < 0.001 (as compared with control cells)

To address whether SDF-1/CXCR7 interaction could mediate in vitro tumor cell-induced tube formation, a coculture system was used in which HUVECs were induced by SGC-7901 cells to form capillary-like structures. The tube formation of HUVECs on the Matrigel was quantified by measuring the tube number. As shown in Fig. 7, control and NC cells induced HUVECs to differentiate into capillary-like structures within 24 h. In contrast, SGC-7901 cells transfected with CXCR7shRNA markedly inhibited tumor cell-induced tube formation. HUVECs showed a significant decrease in the number of tubes after transfecting SGC-7901 with CXCR7shRNA.

### SDF-1 induces VEGF secretion through CXCR7 in SGC-7901 cells

**Fig. 8:**
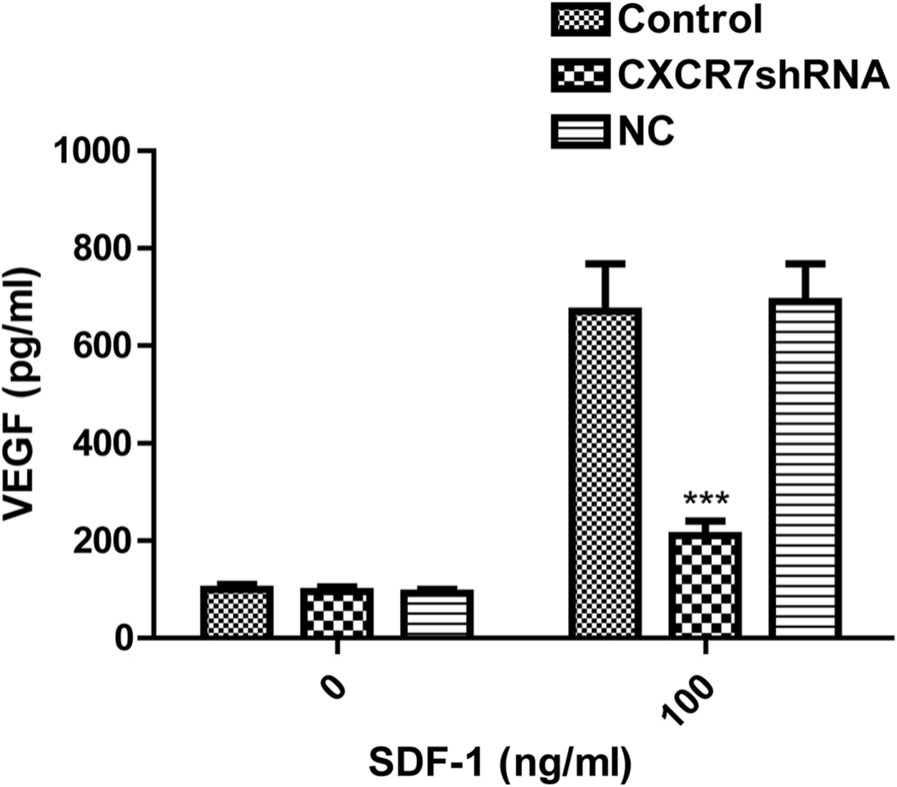
SDF-1 induces VEGF secretion through CXCR7 in SGC-7901 cells. SGC-7901 cells were plated in the 24-well plates. SGC-7901 cells were serum starved for 24 h, and the cells were treated with SDF-1 (0 or 100 ng/ml). The cultured supernatants were harvested 24 h after treatment, and VEGF was measured by ELISA assay. Each *bar* represents mean ± SD from three independent experiments. ***P < 0.001 (as compared with control cells)

To evaluate whether SDF-1 contributes to proangiogenic factor secretion in tumor cells, we treated SGC-7901 cells with SDF-1 and measured the secretion of proangiogenic factor VEGF by ELISA analysis. As shown in Fig. 8, VEGF secretion increased significantly when SGC-7901 cells were treated with SDF-1 for 24 h. To further investigate whether VEGF secretion was mediated by CXCR7, CXCR7 expression was inhibited by RNA interference before treatment with SDF-1. Significant reduction in VEGF secretion was observed in CXCR7shRNA cells compared with control and NC cells. Thus, these findings indicate that SDF-1 can induce VEGF secretion in SGC-7901 cells and that CXCR7 can serve as a factor involved in regulation of secretion of VEGF.

## Discussion

Tumor metastasis is a multistep process that involves the coordinated events of invasion, adhesion, proteolysis, and migration. Considerable efforts have been made in recent years to elucidate the biological function of chemokine receptors in cancer invasion and metastasis. To date, the role of CXCR7 in regulating gastric cancer cell invasion is unclear. In this study, we found that expression of CXCR7 is elevated in all five gastric cancer cell lines. In addition, we observed that treatment with SDF-1 enhanced proliferation and invasion, and silencing of CXCR7 significantly inhibited the proliferation and invasive ability of SGC-7901 cells. Our study indicated the significance of CXCR7 on gastric cancer cell proliferation and invasion. These results are consistent with recent studies showing that CXCR7 mediates chemotaxis of cancer cells toward SDF-1 [21, 26].

Tumor cells interact with ECM components and basement membranes, an essential initial event during the process of invasion. It also has been reported that expression of CXCR7 can regulate adhesion of tumor cells to endothelial cells [14, 27]. Our results demonstrated that SDF-1 could induce adhesion of SGC-7901 cells to FN and LN. The enhanced cell-matrix adhesion may contribute to the metastasis of tumor cells. In addition, we also found that RNA-mediated downregulation of CXCR7 significantly inhibited SDF-1-induced adhesion of SGC-7901 cells to LN or FN. Therefore, these findings clearly indicate that CXCR7 participate in SDF-1-induced cell-matrix adhesion.

Some studies have shown that CXCR7 cannot trigger chemotaxis and activate calcium mobilization and intracellular signaling cascades, such as PI3K and ERK pathways [14, 28]. However, a recent study has demonstrated that CXCR7 is not a decoy but a functional seven-transmembrane span chemokine receptor and can induce phosphorylation of MAPK p42/44 and AKT in human rhabdomyosarcoma cell lines [16]. In this study, we did not elucidate the molecular mechanisms by which CXCR7 regulated the proliferation, adhesion, and invasion of gastric cancer cells. Another recent study suggests that signaling pathways mediated by CXCR7 are independent of those triggered through CXCR4 [29]. Therefore, it is reasonable to speculate that CXCR7 may exert effects on other signaling. Also, the different biological effects elicited by CXCR7 may depend on cell type. Thus, further studies elucidating roles of CXCR7 in invasion and signaling cascades activated by SDF-1/CXCR7 axis are required.

Cancer cells depend on angiogenesis to survive and proliferate. We observed that gastric cancer cells could induce in vitro tube formation, which could promote tumor growth [30]. Although SDF-1-induced VEGF secretion has been reported in various cells, such as lung and prostate cancer cells [21, 31], SDF-1-induced VEGF production in gastric cancer cells has not been previously studied. In the current study, we found that SDF-1/CXCR7 interaction promoted the secretion of VEGF, a potent survival factor for endothelial cells, and one of the most prominent angiogenic factors produced by various tumor cells. Furthermore, our data demonstrate that the knockdown of CXCR7 inhibits secretion of VEGF and tube formation, suggesting that CXCR7 may be involved in the regulation of angiogenesis in gastric cancer.

The above findings imply that SDF-1/CXCR7 interaction may regulate multiple processes in gastric cancer gastric cancer invasion and tumor growth. First, CXCR7 was expressed in all gastric cancer cells. Second, CXCR7 could affect SDF-1-induced tumor cell proliferation, adhesion, and invasion. Third, CXCR7 could regulate gastric cancer invasive ability through angiogenesis and VEGF secretion. Thus, we provide mechanistic evidence that SDF-1/CXCR7 interaction may affect gastric cancer progression by multiple mechanisms including proliferation, adhesion, invasion, angiogenesis, VEGF production, and tumor growth. Because CXCR4 is also a receptor for SDF-1, we cannot exclude the possibility that CXCR4 may be involved in regulating these biological behaviors triggered by CXCR7. Although our study shows the importance of CXCR7 in gastric cancer proliferation, adhesion, invasion, and angiogenesis, the role of SDF-1/CXCR7 interaction in tumor progression are not fully established. Moreover, a recent study has shown that AMD3100, a small synthetic inhibitor of CXCR4, not binds only to CXCR4 but also to CXCR7 [30]. We propose that more attention should be paid to SDF-1/CXCR4 axis and SDF-1/CXCR7 axis. Thus, further studies elucidating the role of SDF-1/CXCR7 axis in cancer development are needed.

## Conclusions

In summary, we presented the first evidence that CXCR7 was expressed in gastric cancer cells. We also observed that suppression of CXCR7 expression by RNA interference impairs in vitro cellular invasion, adhesion, VEGF secretion, and angiogenesis. Taken together, this study provides novel evidence that inhibition of CXCR7 expression may be an effective approach to suppressing tumor growth of gastric cancer.

## Abbreviations

CXCR7: CXC chemokine receptor type 7
ECM: Extracellular matrix
MAPK: Mitogen-activated protein kinase
SDF-1: Stromal cell-derived factor-1
VEGF: Vascular endothelial growth factor
